# Supplementary material for: Identification of photoperiod-induced specific miRNAs in the adrenal glands of Sunite sheep (Ovis aries)
Source: Front Vet Sci. 2022 Jul 22;9:888207. doi: 10.3389/fvets.2022.888207 (PMC9354845; doi:10.3389/fvets.2022.888207)
Supplement: Supplementary file 1 [file Data_Sheet_1.ZIP › Supplementary file/miRDeep2 file.zip!/miRDeep2 file/Know_Structure/SP42A1/oar-mir-3957.pdf]

miRBase precursor : oar-mir-3957
Total read count : 57
oar-miR-3957-5p read count : 47
oar-miR-3957-3p read count : 10
remaining reads : 0

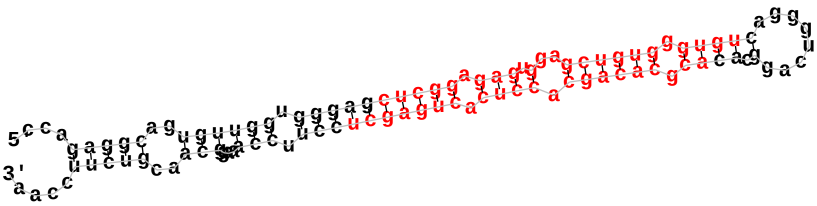

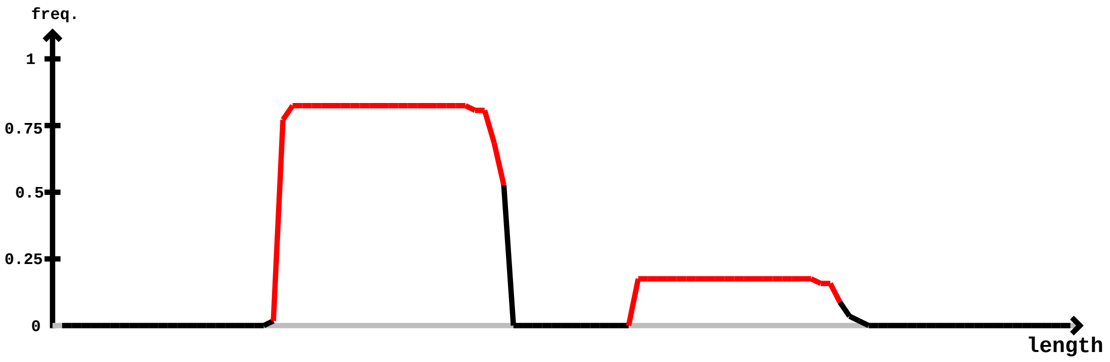


```
                   oar-miR-3957-5p
                                        oar-miR-3957-3p
5'- ccagaggcaguguugguggggagcucggagaguggagcugugggugucagggucaggcacacgcacagcaccucacugagcuccuuccaaaggcaacgucuuccaa   -3'    exp
    ...(((((..((((((.((((((((((.(((.(..((((((.(((((........).)))).)))))).)))).)))))))))).)))...)))..)))))....   reads   mm   sample
    .....................gcucggagaguggagcugugggug............................................................   1       0    seq
    ......................cucggagaguggagcugugg...............................................................   1       0    seq
    ......................cucggagaguggagcugugggu.............................................................   7       0    seq
    ......................cucggagaguggagcugugggug............................................................   7       0    seq
    ......................cucggagaguggagcuguggggugu..........................................................   28      0    seq
    .......................ucggagaguggagcugugggug............................................................   1       0    seq
    .......................ucggagaguggagcuguggggugu..........................................................   2       0    seq
    ...........................................................acgcacagcaccucacuga...........................   1       0    seq
    ...........................................................acgcacagcaccucacugagc.........................   4       0    seq
    ...........................................................acgcacagcaccucacugagcu........................   3       0    seq
    ...........................................................acgcacagcaccucacugagcuc.......................   1       0    seq
    ...........................................................acgcacagcaccucacugagcucc......................   1       0    seq
```
